# The short- to midterm effectiveness of stemless prostheses compared to stemmed prostheses for patients who underwent total shoulder arthroplasty: a meta-analysis

**DOI:** 10.1186/s13018-019-1515-0

**Published:** 2019-12-30

**Authors:** Wei Peng, Yufu Ou, Chenglong Wang, Jianxun Wei, Xiaoping Mu, Zhian He

**Affiliations:** 1 Department of Anatomy and Cell Biology, Justus-Liebig University, 35392 Giessen, Germany; 2 Department of Orthopaedics, The People’s Hospital of Guangxi Zhuang Autonomous Region, Nanning, 530021 China; 3 Department of Orthopaedics, The Affiliated Chencun Hospital of Shunde Hospital, Southern Medical University, Foshan, 528300 China

**Keywords:** Total shoulder arthroplasty, Stemless, Stemmed, Clinical effectiveness, Meta-analysis

## Abstract

**Background:**

To systematically compare the short- to midterm effectiveness of stemless prostheses to that of stemmed prostheses for patients who underwent total shoulder arthroplasty (TSA) and to provide a guideline for clinical decision-making.

**Methods:**

PubMed, the Cochrane Library, and Web of Science were searched with the given search terms until July 2019 to identify published articles evaluating the clinical outcomes for stemless prostheses compared with stemmed prostheses for patients who underwent TSA. Data extraction and the quality assessment of the included studies were independently performed by two authors. Stata software 14.0 was used to analyze and synthesize the data.

**Results:**

Two randomized controlled trials and six case-controlled studies with a total of 347 shoulders were included in this meta-analysis. The results of this meta-analysis showed that there were no significant differences between the stemless and stemmed prostheses in terms of the Constant score, pain score, strength, activities of daily living, postoperative range of motion (ROM), and postoperative maximum active ROM.

**Conclusions:**

This is the first meta-analysis reporting the clinical results of stemless TSA in the short- to midterm follow-up period. Both types of shoulder prostheses were similar in achieving satisfactory clinical outcomes.

## Introduction

Conventional stemmed total shoulder arthroplasty (sTSA) has been considered the standard surgical procedure for patients with primary glenohumeral osteoarthritis because of its outstanding clinical results related to pain relief and restoration of range of motion (ROM), especially in improving patients’ postoperative quality of life [1–3]. However, with the increase in the number of patients who have undergone TSA and the length of follow-up, complications related to the humeral stem, which include intraoperative and postoperative periprosthetic fractures, bone stock loss, malpositioning of the humeral component and metaphyseal stress-shielding, have been reported [4–6].

To reduce the above-mentioned potential risks associated with the humeral stem, a new generation of stemless total shoulder prostheses, such as the Total Evolutive Shoulder System (TESS; Biomet Inc. Warsaw, US), was introduced in 2004 [7]. Subsequently, almost all published studies reported that stemless total shoulder arthroplasty (slTSA) had promising radiological and clinical outcomes in short- to midterm follow-ups [8, 9].

Currently, several published studies [3, 10–16] have compared the differences in the clinical and radiological outcomes between slTSA and sTSA. However, the levels of evidence of these studies were generally limited by small sample sizes and study design. To the best of our knowledge, no previous meta-analysis on this topic was available. Therefore, the purpose of this meta-analysis was to determine whether slTSA was superior to sTSA in terms of clinical and radiological outcomes and to provide guidelines for clinical decision-making.

## Materials and methods

This meta-analysis was performed in strict accordance with the standard methods of the Cochrane Handbook [17]. The preferred reporting protocols for the meta-analyses proposed by the Preferred Reporting Items for Systematic Review and Meta-analysis (PRISMA) Group [18] served as guidelines for reporting the study results.

### Search strategy

The search strategy was designed before the literature search by two authors (W.P. and X.P.M.) with experience in literature retrieval. Then, these two authors independently searched the electronic databases (PubMed, the Cochrane Library, and Web of Science) with the given search terms, which included total shoulder prosthesis, total shoulder replacement, total shoulder arthroplasty, canal-sparing, stemless, and stemmed, for the periods from the establishment of each database to July 2019. Only published works in English were included. The eligible studies were reviewed for relevance; a manual search was also performed on the lists of references to identify those studies that were not included in the preliminary database search.

### Eligibility criteria

The studies meeting the following eligibility criteria were included in this meta-analysis: (i) study population: adult patients with severe shoulder diseases (osteoarthritis, cuff-tear arthropathy, fracture, etc.) who underwent primary TSA; (ii) interventions: slTSA (investigative group) versus sTSA (control group); (iii) outcome indicators (at least one of the following outcome indicators): Constant score, pain score, strength, activity of daily living, mobility, maximum active range of motions (flexion, abduction, intrarotation, extrarotation); and (iv) study design: randomized controlled trial (RCT) or case-controlled study.

The exclusion criteria included the following: (i) incomplete or inappropriate data presentation and (ii) animal experiments, biomechanical studies, reviews, commentaries, conference reports, or case reports.

### Data extraction

A premade standard summary form was filled immediately by two authors (X.P.M. and Y.F. O) after they independently extracted data in accordance with the established protocol. The necessary information was extracted from each study as follows: (i) study characteristics: the name of the first author, publication year, country, study design, sample size, patients’ age and sex, follow-up time, prostheses type, and study population and (ii) outcome indicators: Constant score (pain score, strength, activity of daily living, mobility, maximum active ROM).

### Risk of bias and quality assessment

Two reviewers (W.P. and C.L.W) independently evaluated the quality of each included study using the Newcastle-Ottawa Quality Assessment Scale (NOQAS) for the case-control study and the risk of bias assessment tool proposed by the Cochrane back review group for RCTs. Any controversy was settled by a thorough discussion between the two reviewers.

Low risk, unclear risk, and high risk were assessed for each RCT as proposed by the bias risk assessment tool, which consisted of the following 7-point criteria [17]: (i) random sequence generation (selection bias), (ii) allocation concealment (selection bias), (iii) blinding of participants and personnel (performance bias), (iv) blinding of outcome assessment (detection bias), (v) incomplete outcome data (attrition bias), (vi) selective reporting (reporting bias), and (vii) other bias.

The NOQAS with eight items in three categories was composed of 9 points [19]: 4 points for the selection of the study population (adequate definition of the included cases, representativeness of the cases, selection of controls, definition of controls), 2 points for the comparability between groups (comparability of cases and controls on the basis of the design or analysis), and 3 points for the measurement of exposure factors (ascertainment of exposure, same method of ascertainment for cases and controls, nonresponse rate). We considered a study to be high-quality when its total number of points was greater than or equal to 6 points.

### Statistical analysis

RevMan software 5.2.0 was used to evaluate the risk of bias for RCTs, and we used Stata 14.0 to analyze and synthesize the data. The standardized mean difference (SMD) was used for continuous data with different or unclear scales. Otherwise, the mean difference (MD) was employed. The interpretation of the results relied mainly on the combination SMD/MD and the 95% confidence intervals (95% CI).

The heterogeneity of the results was detected by combining the *Q* test (*a* = 0.10) with the *I*^2^ statistic. The fixed-effect model was employed; when *P* ≥ 0.10 and *I*^2^ ≤ 50%, there was no significant statistical heterogeneity. Otherwise, we used a random-effects model to synthesize the results.

A sensitivity analysis for the outcome indicator that included more than five articles was performed using the one-by-one elimination method to test whether the results were robust. The publication bias of the study results was determined by using funnel plots if there were ten included studies.

## Results

### Study selection

**Fig. 1:**
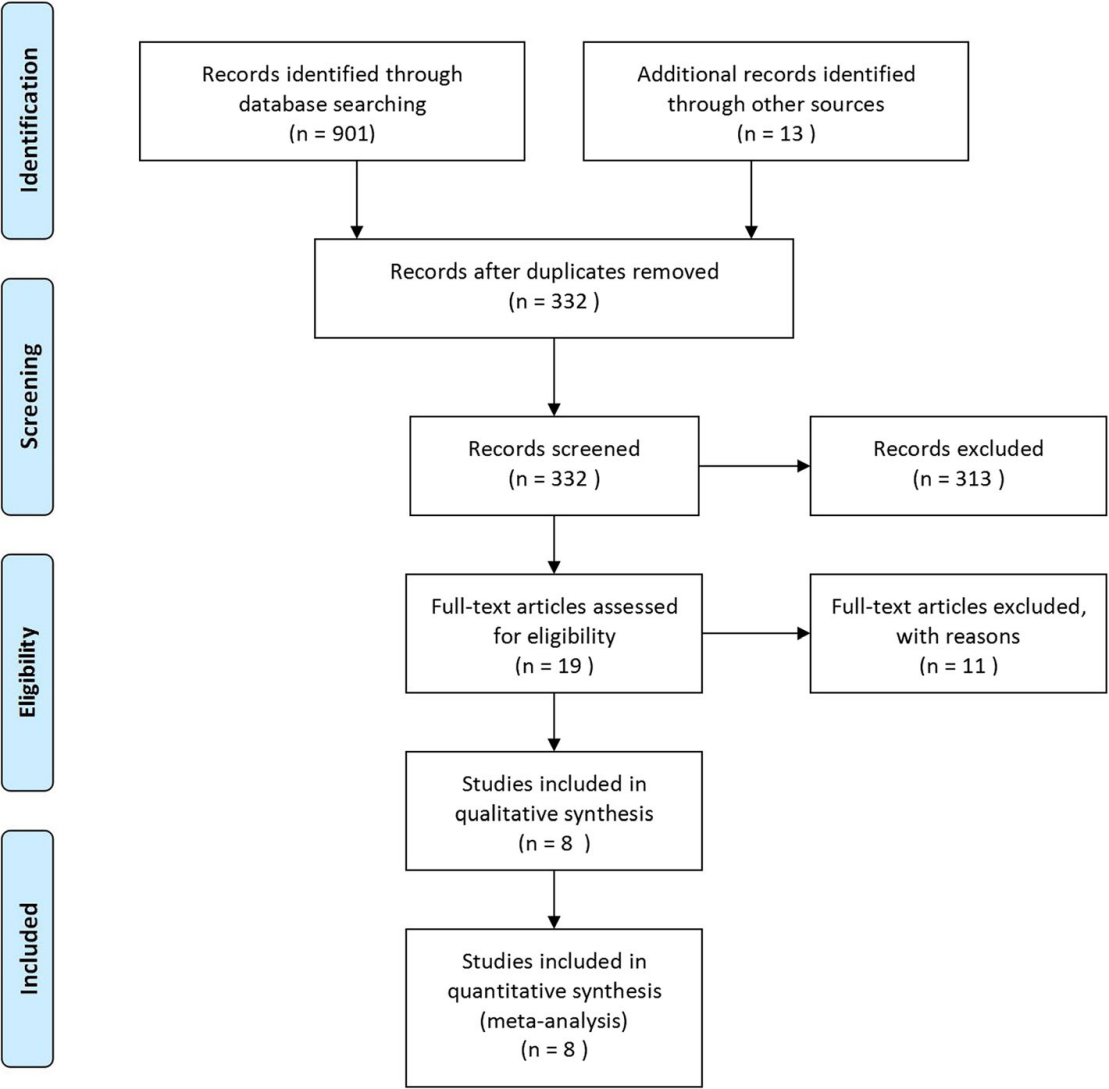
PRISMA 2009 flow diagram

The flow chart under the guidance of the PRISMA statement is shown in Fig. 1. A total of 901 potential studies were preliminarily retrieved in accordance with the predetermined search strategy. A total of 569 duplicated studies were excluded after integration using EndNote (Thomson Corporation, Connecticut, USA). After examining the titles and abstracts, 313 articles were eliminated due to incompatibility with the inclusion criteria. Nineteen studies with full-length texts were identified. Finally, 8 articles [3, 10–16] that met the inclusion criteria were included in this meta-analysis after eliminating 3 studies [9, 20, 21] that did not have complete data.

### Study characteristics and quality assessment

Table 1 shows the characteristics of the included studies. Eight studies with a total of 347 shoulders (investigative group, 172 prostheses; control group, 175 prostheses) were presented in this meta-analysis. Four studies [3, 11, 13, 15] were performed in Germany, and the remaining 4 studies were from Switzerland [14], Austria [12], Italy [10], and Canada [16]. The publication years were from 2013 to 2017.

**Table 1:** Characteristics of the included study

Author (years)	Country	Study design	Sample sizes	Age (years)	Gender (M/F)	Prostheses types	Follow-up (months)	Study population	Outcomes
			slTSA/sTSA	slTSA/sTSA	slTSA/sTSA	slTSA/sTSA	slTSA/sTSA		
Uschok et al. [15]	Germany	RCT	20/20	65/69	10/10; 7/13	Eclipse/Univers II	60/60	Primary glenohumeral OA	①②③④⑤⑥
Glanzmann et al. [14]	Switzerland	CS	37/37	64.4 ± 13/66.7 ± 11.7	9/28; 9/28	Promos/Promos	24/24	Primary glenohumeral OA	②
Spranz et al. [13]	Germany	CS	12/13	74.0 ± 5.7/71.0 ± 5.4	5/6; 4/5	TESS/Aequalis	51.6/75.6	Primary glenohumeral OA	①②③④⑤⑥
Moroder et al. [12[	Austria	CS	24/24	75.6 ± 4.6/74.3 ± 4.6	7/17; 7/17	TESS/Delta Xtend	34.2 ± 10.5/35.2 ± 14.6	Cuff-tear arthropathy	②③④
Maier et al. [11]	Germany	CS	12/12	68.3 ± 5.4/67.8 ± 7.1	7/5; 3/9	TESS/Aequalis	6/6	Primary glenohumeral OA	①②③④⑤⑥
Mariotti et al. [10]	Italy	RCT	9/10	–	–	Aequalis/Aequalis	24/24	Primary concentric OA	②③
Berth and Pap 2013 [3]	Germany	CS	41/41	67.22 ± 9.0/67.05 ± 8.5	14/27; 14/27	TESS/Affinis	30.8 ± 4.6/32.7 ± 4.8	Primary glenohumeral OA	①②③④⑤⑥
Razmjou et al. [16]	Canada	PS	17/18	69.0 ± 9.0/65.0 ± 11.0	8/9; 8/10	TESS/Neer	24/24	Primary glenohumeral OA	②③④

*slTSA* stemless total shoulder arthroplasty, *sTSA* stemmed total shoulder arthroplasty, *M* male, *F* female, *RCT* randomized controlled trial, *PS* prospective study, *CS* case-controlled study; *OA* osteoarthritis; ① pain, ② Constant score, ③ active range of motions (anteversion, abduction, external rotation), ④ strength/power, ⑤ activity, ⑥ motion/mobility

**Fig. 2:**
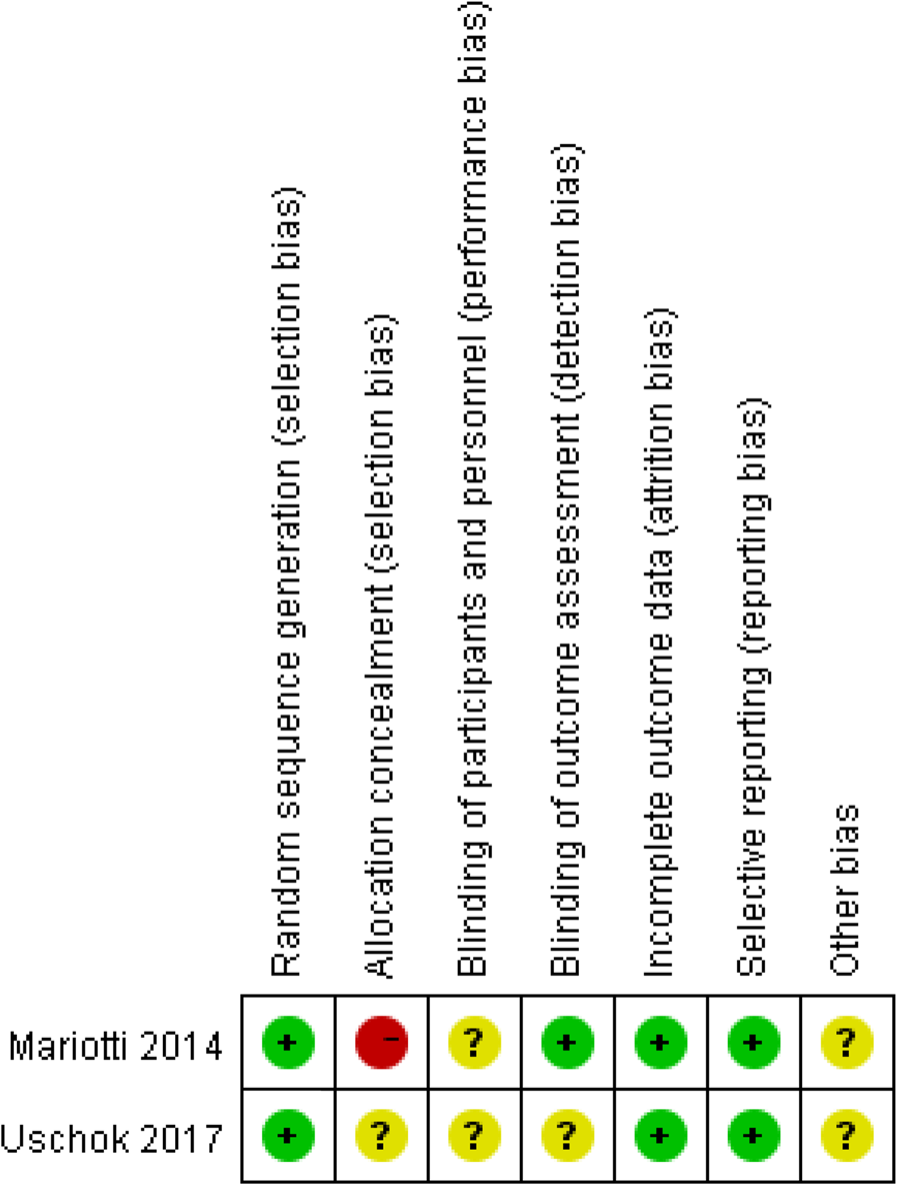
The risk of bias assessment for the included RCTs

The risk of bias assessment for the included RCTs is summarized in Fig. 2, and the quality of the RCTs was rated as moderate. The NOQAS score of each case-controlled study is shown in Table 2. According to the NOQAS, the score of each study was greater or equal to 6 points and was rated as high quality.

**Table 2:** Score distribution of quality assessment based on Newcastle-Ottawa Scale

Items	Selection of study population	Comparability	Outcome evaluation	Total scores
Spranz et al. [13]	☆☆☆	☆	☆☆	6
Glanzmann et al. [14]	☆☆☆☆	☆☆	☆☆	8
Moroder et al. [12]	☆☆☆☆	☆☆	☆☆	8
Maier et al. [11]	☆☆☆	☆☆	☆☆	7
Berth and Pap 2013 [3]	☆☆☆☆	☆☆	☆☆	8
Razmjou et al. [16]	☆☆☆	☆☆	☆☆	7

### Results of the meta-analysis

#### Total constant score

**Fig. 3:**
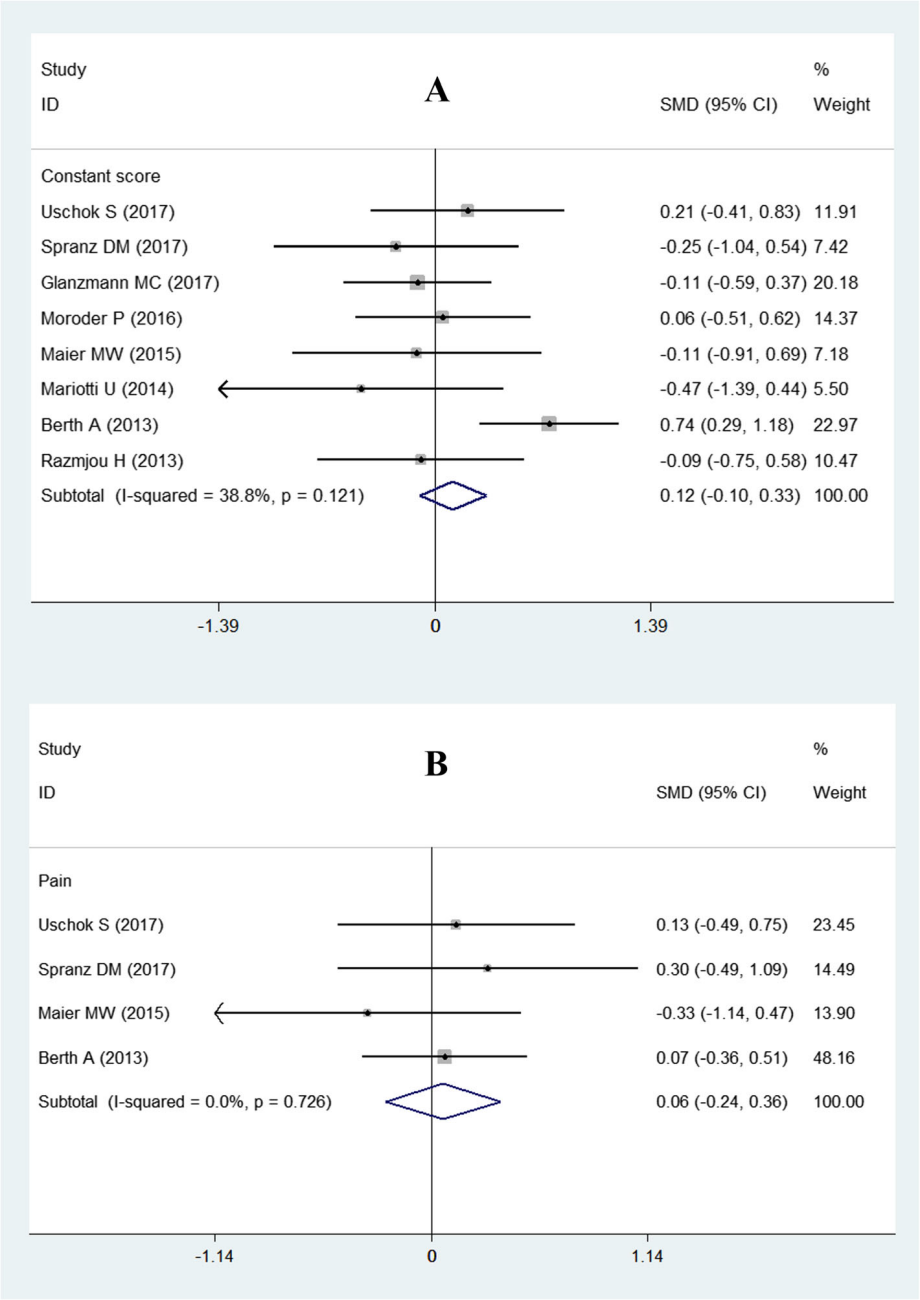
Forest plot for the Constant score and pain score. **a** Constant score; **b** pain score

All of the included studies [3, 10–16], with a total of 341 shoulders (slTSA, 166 shoulders; sTSA, 175 shoulders), reported the results of the total Constant score for both groups. The test for heterogeneity showed that there was no significant difference (*P* = 0.121; *I*^2^ = 38.8%). Thus, a fixed-effects model was employed. The meta-analysis results demonstrated that slTSA was not superior to sTSA for improving clinical effectiveness (SMD = 0.12; 95% CI − 0.10 to 0.33), as shown in Fig. 3.

#### Pain score

Four studies [3, 11, 13, 15], including 171 shoulders (slTSA, 85 shoulders; sTSA, 86 shoulders), reported pain scores for these two shoulder prostheses. A fixed-effects model was used for synthesis of results because there was no statistical heterogeneity (*P* = 0.726 and *I*^2^ = 0%). The pooled results showed that there was no significant difference in the pain scores between the slTSA and sTSA groups (SMD = 0.06; 95% CI − 0.24 to 0.36; Fig. 3).

#### Strength

**Fig. 4:**
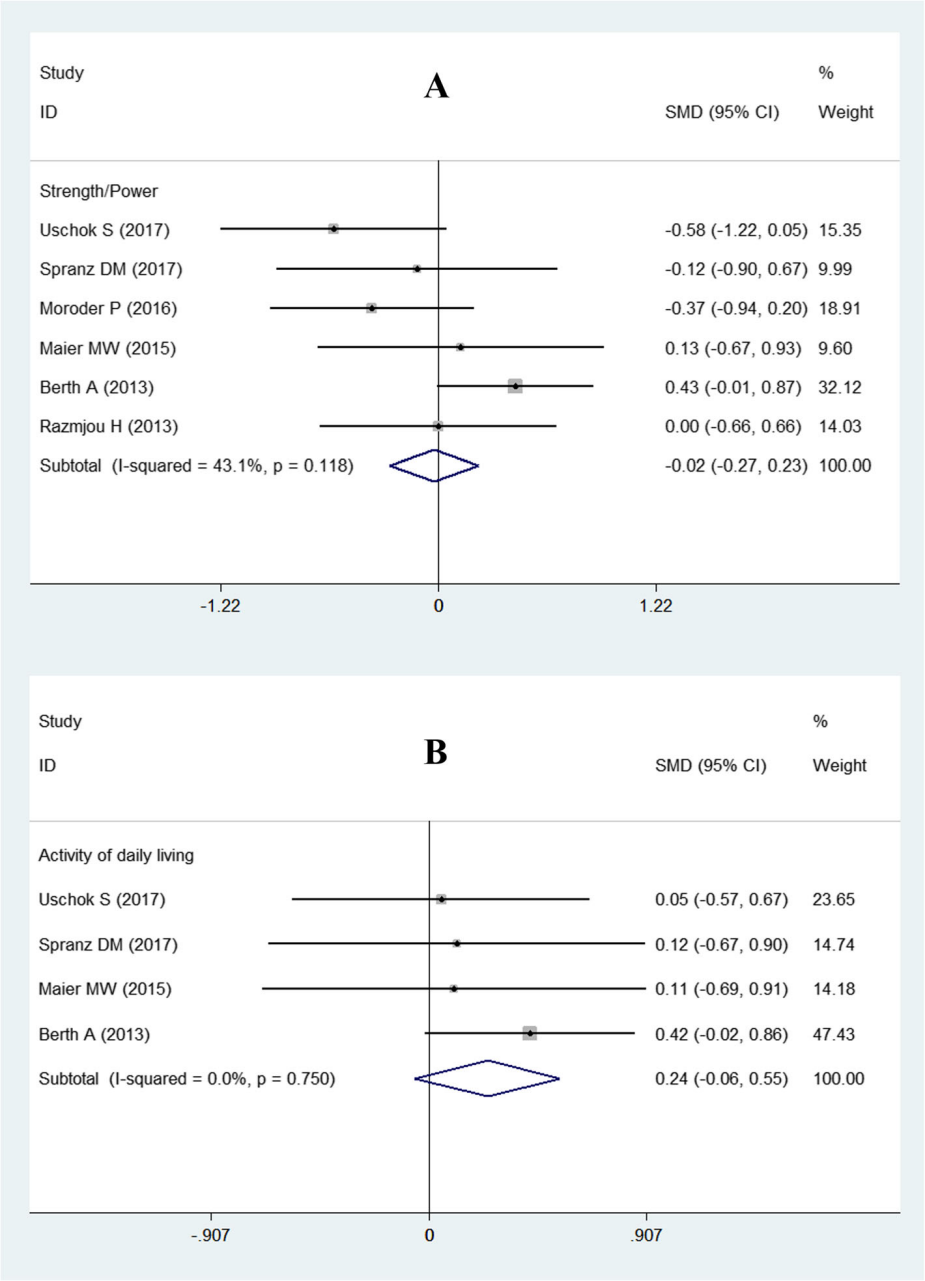
Forest plot for strength and activities of daily living. **a** Strength; **b** activities of daily living

Six studies [3, 11–13, 15, 16] reported the results for strength of the slTSA and sTSA groups, comprising 254 shoulders (slTSA, 126 shoulders; sTSA, 128 shoulders), and the test for heterogeneity was not significant (*P* = 0.118; *I*^*2*^ = 43.1%). Thus, a fixed-effects model was used. The meta-analysis results did not find a significant difference between the two groups (SMD = − 0.02; 95% CI − 0.27 to 0.23; Fig. 4).

#### Activities of daily living

Regarding the activities of daily living, four studies [3, 11, 13, 15] analyzed their improvement after TSA using two different prostheses. We did not detect statistical heterogeneity (*P* = 0.750 and *I*^2^ = 0). Therefore, a fixed-effect model was used, and the pooled results showed no significant difference (SMD = 0.24; 95% CI − 0.06 to 0.55; Fig. 4).

#### Postoperative range of motion (ROM)

**Fig. 5:**
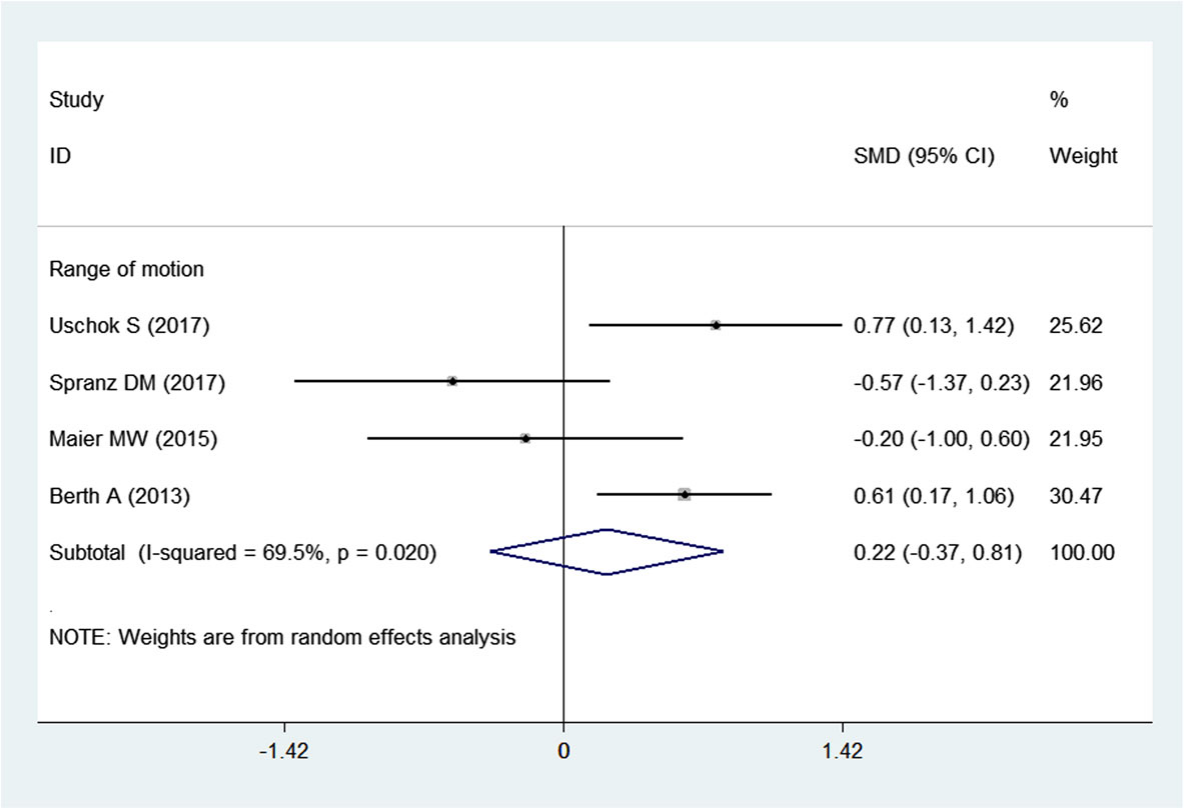
Forest plot for the range of motion (ROM)

Postoperative ROM was reported in four studies [3, 11, 13, 15] with a total of 171 shoulders (slTSA, 85 shoulders; sTSA, 86 shoulders), and slTSA was not better than sTSA in terms of postoperative ROM (SMD = 0.22; 95% CI − 0.37 to 0.81; Fig. 5) after pooled data for the two groups was compared (random-effects model, heterogeneity test: *P* = 0.020 and *I*^2^ = 69.5%).

#### Postoperative maximum active ROM

**Fig. 6:**
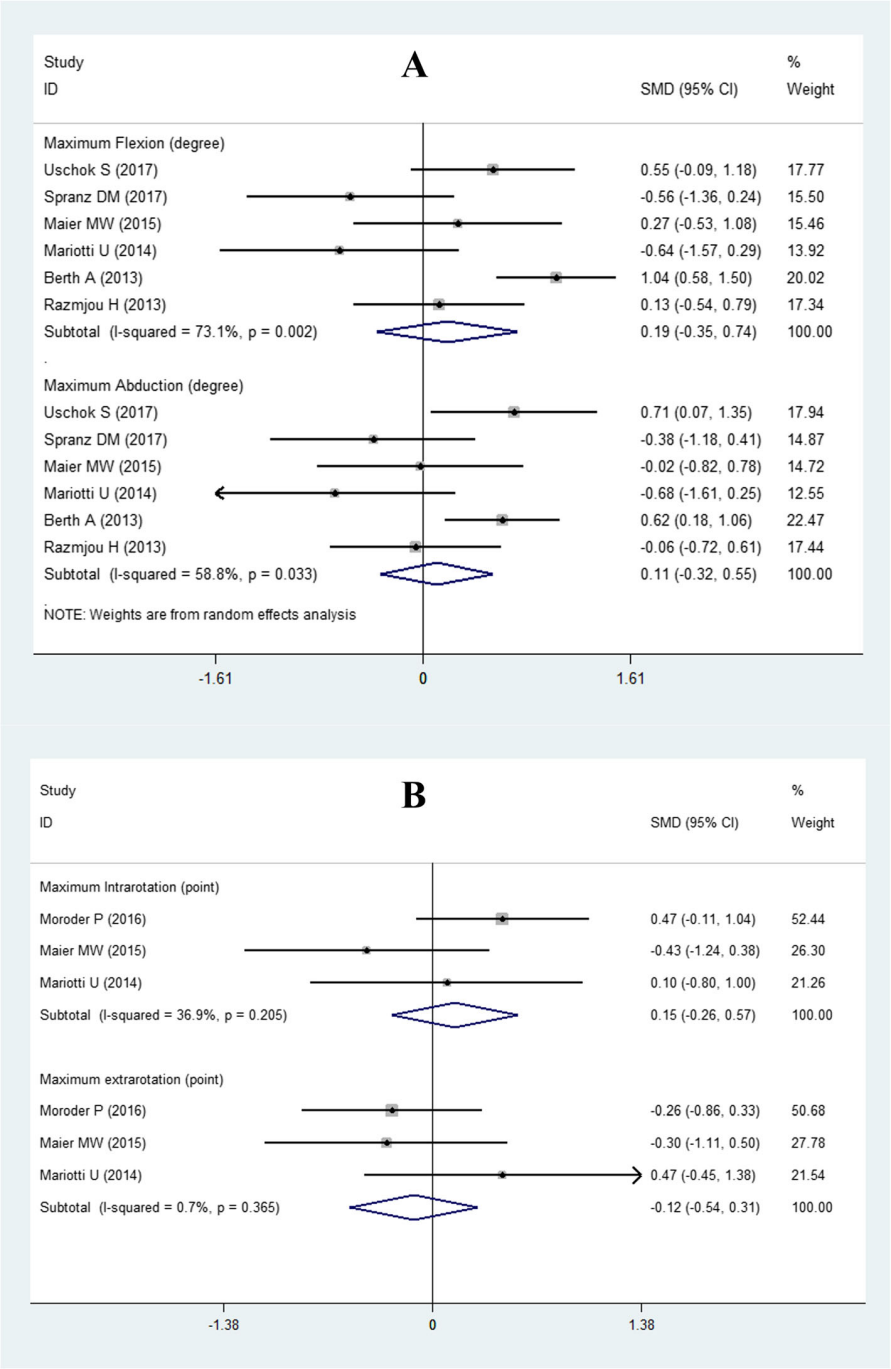
Forest plot for postoperative active ROM. **a** Postoperative maximum flexion and abduction; **b** postoperative maximum internal rotation and external rotation

With regard to maximum active ROM, six studies [3, 10, 11, 13, 15, 16] with 225 shoulders (slTSA, 111 shoulders; sTSA, 114 shoulders) reported the degree of maximum flexion and abduction of the two shoulder prostheses, and there were no significant differences between the two groups (flexion: SMD = 0.19, 95% CI − 0.35 to 0.74; abduction: SMD = 0.11, 95% CI − 0.32 to 0.55, Fig. 6). The random-effects model was employed due to the high statistical heterogeneity (flexion: *P* = 0.002 and *I*^2^ = 73.1%; abduction: *P* = 0.033 and *I*^2^ = 58.8%).

There were three studies [10–12] with sufficient data about the maximum internal rotation and external rotation of the two shoulder prostheses. The fixed-effects model was used for data analysis on the premise of no statistical heterogeneity (internal rotation: *P* = 0.205 and *I*^2^ = 36.9%; external rotation: *P* = 0.365 and *I*^2^ = 0.7%). The pooled results revealed that there were no significant differences in maximum internal rotation (SMD = 0.15; 95% CI − 0.26 to 0.57; Fig. 6) and external rotation (SMD = − 0.12; 95% CI − 0.54 to 0.31; Fig. 6).

#### Sensitivity analysis and publication bias

The outcome indicators that were included in more than five studies were required to undergo a sensitivity analysis. These outcome indicators were as follows: Constant score, strength/power, maximum flexion, and abduction. We detected that the pooled results for maximum flexion could not be considered robust due to the existence of a significant difference with the one-by-one elimination method (SMD = 0.39, 95% CI 0.12 to 0.66). A publication bias was not performed because there were not enough articles (in general, more than ten studies).

## Discussion

Currently, slTSA is a popular surgical procedure for patients with severe shoulder diseases. However, there is still controversy regarding whether slTSA is superior to sTSA. To the best of our knowledge, this is the first meta-analysis aimed at comparing the short- to midterm effectiveness of stemless and stemmed prostheses for patients who underwent TSA. The results of this meta-analysis revealed that both slTSA and sTSA can improve pain relief and restore the ROM of the shoulder postoperatively in the short and midterm follow-up period. However, the current findings did not support the hypothesis that a stemless prosthesis was a better replacement component for patients who underwent TSA compared with a stemmed prosthesis.

To date, slTSA has been used for over ten years and has experienced an increase in popularity among surgeons [5], but studies on its clinical results are still rare. A recent systematic review of 11 retrospective studies involving 929 cases revealed that all of the published studies of canal-sparing stemless shoulder arthroplasty reported promising clinical and radiological outcomes in the short-to-midterm follow-up period [8]. Patients with TSA might obtain more benefits from the stemless prosthesis, but the clinical choice of this system still requires that the proximal humerus has sufficient bone mass and good bone mineral density [3, 22].

Compared with sTSA, slTSA has several theoretical advantages. First, a stemless prosthesis is helpful to restore the anatomical structure of the proximal humerus, and it is also easier to operate [23]. In the previous literature, the incidence of stem-related complications for slTSA was only 0–7.9% [8]. Using this system seems to preserve more metaphyseal bone stock [15] and to effectively reduce complications related to component removal [24] in revision surgery after TSA. Therefore, we speculated that a stemless prosthesis could achieve better functional outcomes than a stemmed prosthesis.

However, the results of the present meta-analysis overturned our previous hypothesis. Based on the present findings, we could not conclude that the stemless prosthesis should have a higher priority in clinical applications. However, the clinical outcomes of the stemless prosthesis were as good as those of the stemmed prosthesis in the short- to midterm follow-up period and showed that the stemless prosthesis is a great alternative and effective biomaterial for TSA. Additionally, these clinical data may be useful for surgeons in clinical practice because the surgeons prefer to preserve as much bone tissue as possible, shorten the operation time, and reduce postoperative complications. It is possible that further well-conducted and adequately powered clinical studies will demonstrate favorable clinical outcomes following TSA with stemless prostheses [21].

The level of evidence of the meta-analysis is probably level III or lower because of the bias in the included studies. As with other studies, several limitations in the present study have forced us to interpret the results carefully. First, the conclusions of this meta-analysis were significant but incomplete due to a lack of relevant long-term follow-up studies. Moreover, the types of shoulder prostheses and the follow-up time could not be stratified because of the restriction of the small study sample, so the results of several outcome indicators after pooling had high statistical heterogeneity. In addition, the experiences of surgeons and evaluators may also negatively affect the reliability and stability of the results. There may be publication bias and selection bias because we only included studies that were written in English and publicly published. However, the above bias could not be detected well because the meta-analysis had less than ten included studies. Considering the small sample sizes and inconsistent study design of the present study, multicenter, large-sample, well-designed studies are expected to further examine the reliability of the above findings.

## Conclusions

The conclusion that both types of shoulder prostheses achieve consistently good clinical and radiologic outcomes in the short- to midterm follow-up period indicates that clinicians should preferably select the shoulder prostheses for the appropriate patient. However, to obtain more comprehensive results on slTSA, well-designed, multicenter, large-sample, long-term follow-up studies are required.

## Data Availability

The datasets generated and analyzed during the current study are available from the corresponding author on reasonable request.

## Abbreviations

95% CI: 95% confidence intervals
MD: Mean difference
NOQAS: Newcastle-Ottawa Quality Assessment Scale
PRISMA: Preferred Reporting Items for Systematic Review and Meta-analysis
RCT: Randomized controlled trial
ROM: Range of motion
slTSA: Stemless total shoulder arthroplasty
SMD: Standardized mean difference
sTSA: Stemmed total shoulder arthroplasty
TSA: Total shoulder arthroplasty
